# β2-Adrenoceptors in the Medial Prefrontal Cortex Excitatory Neurons Regulate Anxiety-like Behavior in Mice

**DOI:** 10.3390/ijms23105578

**Published:** 2022-05-17

**Authors:** Zhuogui Lei, Yukyan Lam, Cheukhin Li, Zhongqi Fu, Aruna S. Ramkrishnan, Shu Liu, Ying Li

**Affiliations:** 1 Department of Neuroscience, City University of Hong Kong, Hong Kong 999077, China; zhuogulei2-c@my.cityu.edu.hk (Z.L.); zhongqifu2@cityu.edu.hk (Z.F.); aramkrish3@cityu.edu.hk (A.S.R.); sliu93-c@my.cityu.edu.hk (S.L.); 2 Department of Biomedical Sciences, City University of Hong Kong, Hong Kong 999077, China; yukyanlam5-c@my.cityu.edu.hk (Y.L.); chli57-c@my.cityu.edu.hk (C.L.); 3 Centre for Regenerative Medicine and Health, Hong Kong Institute of Science & Innovation, Chinese Academy of Sciences, Hong Kong 999077, China; 4 Centre for Biosystems, Neuroscience, and Nanotechnology, City University of Hong Kong, Hong Kong 999077, China

**Keywords:** norepinephrine, β2-adrenoceptors, optogenetics, neuronal manipulation, microRNA-based silencing, medial prefrontal cortex, excitatory neurons, anxiety, social behavior, anxiolytic therapy

## Abstract

The medial prefrontal cortex (mPFC) and β-adrenoceptors (βARs) have been implicated in modulating anxiety-like behavior. However, the specific contributions of the β2-AR subtype in mPFC in anxiety are still unclear. To address this issue, we used optogenetic and microRNA-based (miRNA) silencing to dissect the role of β2-AR in mPFC in anxiety-like behavior. On the one hand, we use a chimeric rhodopsin/β2-AR (Opto-β2-AR) with in vivo optogenetic techniques to selectively activate β2-adrenergic signaling in excitatory neurons of the mPFC. We found that opto-activation of β2-AR is sufficient to induce anxiety-like behavior and reduce social interaction. On the other hand, we utilize the miRNA silencing technique to specifically knock down the β2-AR in mPFC excitatory neurons. We found that the β2-AR knock down induces anxiolytic-like behavior and promotes social interaction compared to the control group. These data suggest that β2-AR signaling in the mPFC has a critical role in anxiety-like states. These findings suggest that inhibiting of β2-AR signaling in the mPFC may be an effective treatment of anxiety disorders.

## 1. Introduction

Anxiety disorders constitute the most common psychiatric illness, with lifetime prevalence and steadily increasing occurrence [1,2]. Norepinephrine (NE) and β-adrenoceptors (β-ARs) play a critical role in emotional arousal and stressful events [3]. Propranolol, a β-AR antagonist with equal affinity for both β1- and β2-AR [4], was investigated as a general anxiolytic treatment of post-traumatic stress disorder (PTSD), one of the most common anxiety disorders [5,6]. Rodent studies showed that stress-induced anxiety-like behavior, microglial reactivity, and neuronal activation could be reversed by systemic injection of propranolol [7]. In addition, the β2-AR (ADRB2) gene was found to be strongly associated with generalized anxiety disorder (GAD) in the elderly [8]. ADRB2 gene polymorphism was found to be strongly associated with the development of PTSD symptoms in persons with a history of childhood adversity, indicating that the decreased expression of ADRB2 may protect against negative biological consequences of chronic activation of noradrenergic systems in adversity exposure [9]. However, there are a lack of studies about how downregulation of β2-AR in specific brain areas affects anxiety-like behavior.

The locus coeruleus (LC) is the major producer of NE in the brain and LC activation triggers NE release to the basolateral (BLA), the central nucleus of the amygdala (CeA), and the cortex [10,11,12,13]. Optogenetic activation of LC induced anxiety-like behavior which can be reversed by systemic blockade of β-ARs antagonists instead of α1-ARs antagonists [14]. The Siuda group found that optogenetic activation of BLA β2-AR induced acute and social anxiety-like behavior in mice, indicating that activating β2-AR in BLA had an anxiogenic effect [15]. However, using the β2-AR knockout mice model, the Zhu group proved that the deletion of β2-AR increased anxiety and depression levels in mice. Given that an anxiogenic effect can be induced by activating β2-AR in BLA or systemic knockout of β2-AR, understanding how β2-AR in other brain areas regulates anxiety-like behavior is important and may have significant clinical implications.

Apart from the hippocampus and the amygdala, the medial prefrontal cortex (mPFC) also plays a critical role in anxiety and fear [16,17,18,19,20,21]. It has been reported that optogenetic inhibition of the prelimbic (PL) PFC excitatory neurons initiated anxiety-like behavior, while optogenetic activation of PL excitatory neurons had no effect on anxiety-like behavior [21]. Fuchikami demonstrated that optogenetic activation of infralimbic (IL) PFC induced a rapid and sustained anxiolytic effect. However, other groups have reported contradictory results, such as activating IL by local perfusion of bicuculline [22], or optogenetic activation IL excitatory neurons inducing anxiety-like behavior in open field test [23]. Thus, the role of mPFC neurons in anxiety-like behavior is currently under debate.

Chemogenetic activation of LC to mPFC neurons instead of LC to spinal cord neurons induced aversive and anxiety-like behavior [10], indicating that the LC-NE signal to mPFC may have an anxiogenic effect. It is reported that β2-AR are abundantly expressed in mPFC excitatory neurons and activation of mPFC β2-AR enhances long-term potentiation and trace fear memory [24]. Also, the Ramos group found that activating β2-AR in mPFC by local perfusion of clenbuterol, a β2-AR agonist, enhances working memory performance in aging animals [25]. However, the mechanism through which β2-AR in mPFC may regulate anxiety-like behavior is largely unknown.

To dissect the roles of mPFC β2-AR in anxiety-like behavior, we recruit optogenetics to specifically manipulate β2-AR activity [26,27] and miRNA based silencing to knock down β2-AR expression within the pyramidal neurons of the mPFC. Our data show that optogenetic activation of β2-AR is sufficient to induce anxiety-like behavior and β2-AR knock down can induce an anxiolytic effect. Our finding suggests that β2-AR in mPFC may be a potential target for the treatment of anxiety disorders.

## 2. Results

### 2.1. Optogenetic Activation of β2-AR in mPFC CaMKIIα Neurons Induced Anxiety in Open-Field Test and Elevated Zero Maze

System blockage of β-adrenergic receptors can reverse the anxiogenic effects induced by optogenetic activation of LC NE neurons [14]. In addition, chemogenetic activation of mPFC projecting LC neurons instead of spinal cord projecting LC neurons can induce an anxiety-like state [10]. Here, we hypothesize that β2-AR in mPFC may be involved in anxiety-like behavior.

**Figure 1:**
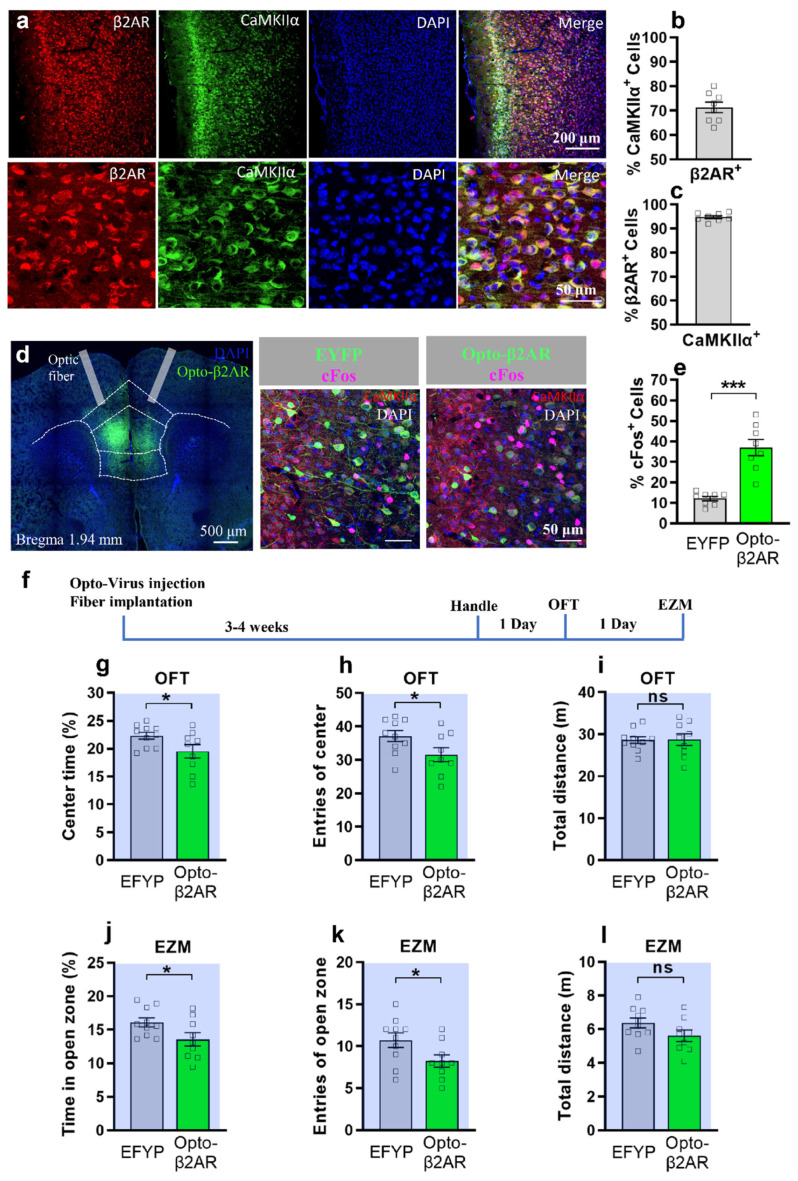
Optogenetic activation of β2AR in mPFC CaMKIIα neurons induces anxiety-like behavior in OFT and EZM. (**a**) Immunofluorescence images showed co-expression with β2-AR (red) and CaMKII(green) in the mPFC. Scale bar: 200, 50 μm. (**b**,**c**) Quantification of percentage of β2-AR positive cells co-expressed with CaMKIIα, and percentage of CaMKIIα positive cells co-expressed with β2-AR. (**d**) Bilateral viral injection sites and optic fiber implants in the mPFC (left panel, scale bars: 50 μm). Opto-β2-AR slice showed more cFos (Pink) expression than EYFP slice after light stimulation (right panel, scale bars: 50 μm). (**e**) Quantification of percentage of EYFP or β2-AR positive cells co-expressed with cFos, unpaired student *t*-test, *** *p* < 0.001. (**f**) Behavior Testing Timeline. (**g**–**i**) Opto-β2AR^PFC/CaMKIIα^ mice (green) spend less time (*p* = 0.0483) and had fewer entries (*p* = 0.0495) of center in the open field as compared with control (grey) (unpaired-*t*-test, * *p* < 0.05). No significant difference is shown in the total distance (unpaired-*t*-test, ns, *p* = 0.9396). (**j**–**l**) Opto-β2AR^PFC/CaMKIIα^ mice (green) spend less time in center (*p* = 0.0494) and had fewer entries of open zone (*p* = 0.0469) in the elevated zero maze as compared with control (grey) (unpaired student *t*-test, * *p* < 0.05). No significant difference is shown in the total distance (unpaired-*t*-test, ns, *p* = 0.100, opto-β2AR group, *n* = 9, control group, *n* = 10).

First, to test whether β2-AR are expressed abundantly in mPFC, which consists of anterior cingulate cortex (ACC), prelimbic cortex (PL), and infralimbic cortex (IL) [28], we use immunohistochemistry to stain β2-AR and CaMKIIα, the excitatory neuron marker [29]. We found that 71.34 ± 2.15% β2-AR positive cells are co-expressed with CaMKIIα, while 94.79 ± 0.60% CaMKIIα positive cells are co-expressed with β2-AR (Figure 1a–c). To confirm the specificity and expression pattern of β2-AR in mFPC, we used new β2-AR antibody from another company to co-stain with CaMKIIα and found a similar result (Supplementary Figure S1a). Then, to specifically manipulate mPFC β2-AR in real time, we utilized β2-AR optogenetics, which is similar in dynamics to endogenous β2-AR [26,27].

To test whether opto-activation of β2-AR in mPFC excitatory neurons can induce anxiety-like behavior, cre dependent AAV-ef1a-Dio-opto-β2-AR-EYFP or control virus together with AAV-CaMKIIα-cre were bilaterally injected into the mPFC and optical fibers were implanted (Figure 1d). To validate the activation effect of opto-β2-AR, we examined the expression of immediate early gene c-Fos after delivery of light (473 nm, 10 Hz, pulse 10% duty, 8–10 mW power) to mice via optic fiber three to four weeks after virus injection. We found that opto-β2-AR-EYFP neurons showed a highly significant increase of co-expression with c-Fos compared to EYFP neurons after light stimulation (Figure 1d,e). Also, the opto-β2-AR-EYFP neurons have 98.45 ± 0.43% co-expression with CaMKIIα (Supplementary Figure S1b,c). These data indicate that opto-β2-AR can induce an excitatory effect on neurons with high cell specificity. Then, using another group of animals injected with the virus (Figure 1f, Supplementary Figure S2a), we used the open field test (OFT) to study the role of mPFC opto-β2-AR in acute anxiety-like behavior. Three weeks after virus injection, Opto-β2-AR mice and control mice received the same optogenetic stimulation (473 nm, 10 Hz, pulse 10% duty, 8–10 mW power) continuously for 6 min in the OFT via optic fiber. Opto-activation of β2-AR/CaMKIIα in the mPFC produced anxiogenic-like behavior as the opto-β2-AR/CaMKIIα mice (*n* = 9) spent significantly less time or entered less in the center of the OFT compared to EYFP controls (*n* = 10) during optogenetic activation (Figure 1g,h). Also, we observed no differences in total distance traveled in both controls and opto-β2-AR/CaMKIIα animals, indicating that opto-activation of β2-AR/CaMKIIα had no effect on animal mobility (Figure 1i). Then, we utilize the elevated zero maze (EZM) to further test the anxiogenic effect of optogenetic activation of mPFC β2-AR. We found that the opto-β2-AR/CaMKIIα group showed decreased time and entries in the open zone, compared to the control group (Figure 1j,k), while the total distance showed no difference (Figure 1l). Also, the opto-β2-AR/CaMKIIα group showed no significant difference in the time or entries in the center of the OFT (Supplementary Figure S3a) or in the open arms of the EPM (Supplementary Figure S3b) after optogenetic activation. This data suggests that opotogenetic activation of β2AR in mPFC excitatory neurons (6 min stimulation) induces acute anxiety-like behavior only during optogenetic stimulation. However, whether opto-activation of β2AR in mPFC for a longer period (e.g., 30 min) can induce a long-lasting effect in anxiety-like behavior has not been addressed in this study and requires future investigation.

### 2.2. Optogenetic Activation of β2-AR within mPFC CaMKIIα Neurons Induced Anxiety-like Behavior in Novelty-Suppressed Feeding Test and Depression-like Behavior in Tail-Suspension Test

**Figure 2:**
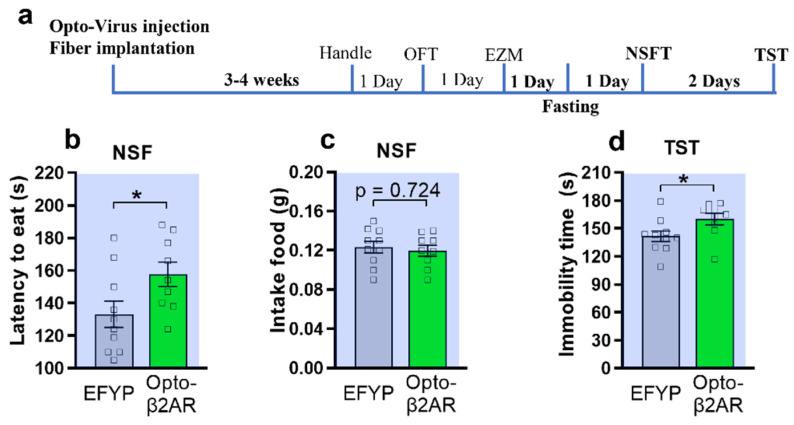
Optogenetic activation of β2AR in mPFC CaMKIIα neurons induces anxiety-like behavior in NSFT and depression-like behavior in TST. (**a**) Behavior Testing Timeline of the NSFT and TST. (**b**,**c**) Effect of AAV-opto-β2AR injections in NSFT in terms of the latency to eat (**b**) and the weight of food intake after the test in 5 min (**c**). (**b**) Opto-β2AR^PFC/CaMKIIα^ mice (green) show increased latency (*p* = 0.0417) as compared with control (grey) (unpaired-*t*-test, * *p*< 0.05,). (**c**) Opto-β2AR^PFC/CaMKIIα^ mice (green) show no significant different in the weight of food intake as compared with control (grey) (unpaired-T test, *p* = 0.724). (**d**) Opto-β2AR^PFC/CaMKIIα^ mice had higher immobility time as compared with control (grey) in the TST (*p* = 0.0462, unpaired-T test, * *p* < 0.05, opto-β2AR group, *n* = 9, control group, *n* = 10).

To further confirm the anxiogenic effect induced by opto-activation of β2-AR within mPFC CaMKIIα neurons, we used a novelty-suppressed feeding test (NSFT), which is a measure of anxiety that is responsive to chronic administration of typical antidepressants [30,31]. After 24 h fasting, the same group of mice performed the NSFT with optogenetic activation (Figure 2a). The opto-β2-AR/CaMKIIα mice showed a significant increase of latency to eat (157.7 ± 7.5) compared to the control group (133.2 ± 8.1, unpair Student *t*-test, Figure 2b). Also, after the NSFT there was no significant difference in total food consumption in the homecage (Figure 2c). To test whether opto-activation of β2-AR within mPFC CaMKIIα neurons can induce depression-like behavior, we used the tail-suspension test (TST) [31] with optogenetics. TST data showed that the opto-β2-AR/CaMKIIα group has significantly increased immobility time compared to the control, indicating that optogenetic manipulation of β2-AR in mPFC could induce depression-like behavior.

### 2.3. Optogenetic Activation of β2-AR within mPFC CaMKIIα Neurons Was Sufficient to Reduce Social Interaction

β-adrenergic receptors have also been implicated in social anxiety disorders and β-blockers are commonly prescribed as anxiolytics in the clinic [32]. Given that studies have shown that the mPFC is critical for social behaviors [19], we hypothesize that activation of β2-AR signaling in mPFC would affect social interaction.

**Figure 3:**
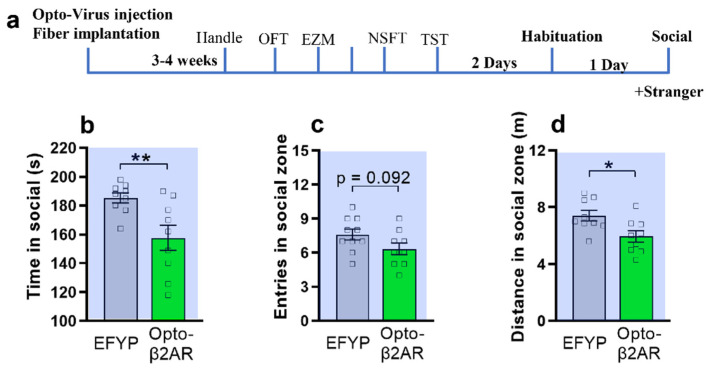
Optogenetic activation of β2AR in mPFC CaMKIIα neurons decrease social interaction. (**a**) Behavior testing timeline of social interaction test. (**b**) Opto-β2AR^PFC/CaMKIIα^ mice (green) spend less time (*p* = 0.0092) in social as compared with control (grey) (unpaired-T test, ** *p*< 0.01). (**c**) Opto-β2AR^PFC/CaMKIIα^ mice (green) had no significant change of entries in social zone as compared with control (grey) (unpaired-T test, *p* = 0.092). (**d**) Opto-β2AR^PFC/CaMKIIα^ mice (green) travel less distance in social zone as compared with control (grey) (*p* = 0.0166, unpaired-T test, * *p*< 0.05, opto-β2AR group, *n* = 9, control group, *n* = 10).

In addition to acute anxiety, we test how optogenetic activation of mPFC β2-AR affect the social interaction in mice in the three-chamber social interaction test (Figure 3a). The mice were habituated in the three chambers. Both opto-β2-AR/CaMKIIα and control mice spent similar amounts of time in the social zone in the absence of a novel conspecific (Supplementary Figure S3c). One day after the habituation, in the presence of a novel mouse and while receiving opto-stimulation, EYFP control animals showed a significant increase in the time spent in the social zone, whereas opto-β2-ARBLA/CaMKIIα animals did not display such social interaction behavior (Figure 3b). In addition, the optogenetic group showed a significant reduction of the distance in the social zone compared to the control group (Figure 3d). Interestingly, there were no differences in number of entries in the social zone, suggesting that opto- β2-AR/CaMKIIα-expressing animals entered the social zone but did not remain there for a long period of time (Figure 3c).

### 2.4. miRNA Silencing of β2-AR within mPFC CaMKIIα Neurons Reduced Anxiety-like Behavior

β2-AR (ADRB2) gene is found to be strongly associated with GAD in the elderly [8]. Inhibiting the ADRB2 may protect against negative biological consequences of chronic activation of noradrenergic systems in adversity exposure [9]. Given that optogenetic activation of β2-AR in mPFC induced an anxiogenic effect, we hypothesized that miRNA silencing of β2-AR signaling in mPFC would induce the anxiolytic effect.

**Figure 4:**
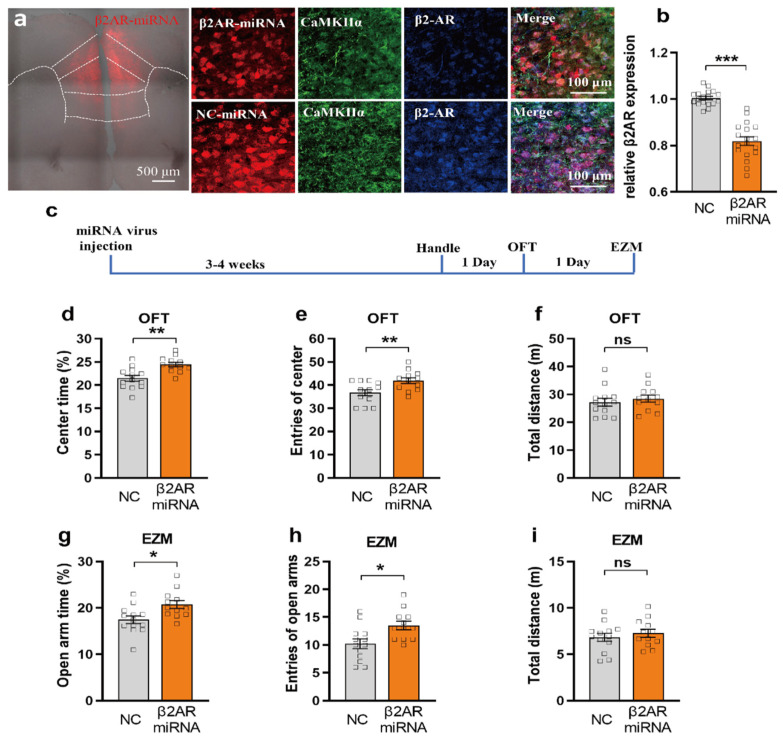
miRNA knock down of β2AR in mPFC CaMKIIα neurons reduces anxiety in OFT and EZM. (**a**) Co-localization β2AR (blue) and CaMKIIα(green) with the β2AR-miRNA(red) in the mPFC. Scale bar: 100μm. Upper panel shown that the β2AR-miRNA results in less expression of β2AR compared to the NC-miRNA (down panel). (**b**) The relative β2AR expression (florescent intensity) was reduced by β2-AR-miRNA (orange) as compared with control (grey) (unpaired-T test, *** *p* < 0.001, *n* = 6, 3 slice each mouse). (**c**) Behavior testing timeline of OFT and EZM. (**d**–**f**) β2AR-miRNA^PFC/CaMKIIα^ mice (orange) spend more time (*p* = 0.0011) and had more entries (*p* = 0.0079) of center in the open field as compared with control (grey) (unpaired-T test, ** *p* < 0.01). No significant difference is shown in the total distance (*p* = 0.5094). (**g**–**i**) β2AR-miRNA^PFC/CaMKIIα^ mice (orange) spend more time (*p* = 0.0105) in open arm and had more entries (*p* = 0.0101) of open arm in the elevated zero maze as compared with control (grey) (unpaired-T test, * *p* < 0.05). No significant difference is shown in the total distance (*p* = 0.4789) (miRNA group *n* = 12, control group *n* = 13), ns, *p* = 0.100.

To specifically knockdown the expression of β2-AR in mPFC pyramidal neurons, we inject the Cre dependent virus of β2-AR-miRNA-mcherry with AAV-CaMKIIα-cre into mPFC [28]. After 3–4 weeks for virus expression, mice were sacrificed and stained for β2-AR and CaMKIIα. β2-AR-miRNA-mcherry is expressed mainly in CaMKIIα neurons, indicating that this double-virus injection has high specificity in pyramidal neurons (Figure 4a). Quantifying the fluorescent intensity of β2-AR expression, we found that the expression level of β2-AR in β2-AR-miRNA-mcherry group is significantly decreased compared to the negative control (NC) group (Figure 4a,b, *n* = 6 mice).

To test the anxiolytic effect by miRNA silencing of β2-AR in mPFC, using another cohort of mice, we injected above AAV-ef1a-Dio-miRNA-β2AR-mcherry or control virus together with AAV-CaMKIIα-cre into mPFC and conducted the OFT and EZM 3–4 weeks later (Figure 4c, Supplementary Figure S2b). We found that miRNA-β2-AR/CaMKIIα (*n* = 12) mice spent significantly more time in or displayed a greater tendency to enter the center of the OFT than EYFP controls (*n* = 13) (Figure 4d,e), suggesting that miRNA silencing can induce an anxiolytic effect. Also, we observed no effect on animal mobility as both controls and miRNA-β2-AR/CaMKIIα animals showed no differences in total distance traveled (Figure 4f, unpaired Student’s *t*-test; *p* = 0.5094). Then, we utilize the EZM to further test the anxiolytic effect of miRNA silencing of mPFC β2-AR. We found that the miRNA-β2-AR/CaMKIIα group showed increased time and entries in the open zone compared to the control group (Figure 4g,h, *p* = 0.0105, and 0.0101), while the total distance showed no difference (Figure 4i).

### 2.5. miRNA Silencing of β2-AR within mPFC CaMKIIα Neurons Induced Anxiolytic-like Behavior in NSFT and Antidepression-like Behavior in TST

**Figure 5:**
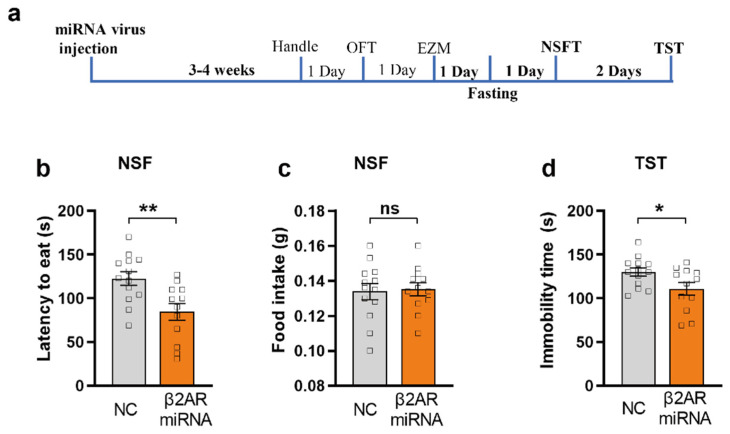
miRNA knock down of β2AR in mPFC CaMKIIα neurons reduces anxiety in NSFT and depression-like behavior in TST. (**a**) Behavior Testing Timeline of NSFT and TST. (**b**,**c**) Effect of AAV-β2AR-miRNA injections in NSFT in terms of the latency to eat (**b**) and the weight of food intake (**c**). (**b**) β2AR-miRNA^PFC/CaMKIIα^ mice (orange) show decreased latency as compared with control (grey) (unpaired-T test, *p* = 0.0049, ** *p* < 0.01). (**c**) β2AR-miRNA^PFC/CaMKIIα^ mice (orange) and control (grey) show no difference in weight of food intake (*p* = 0.8173) after the NSF. (**d**) β2AR-miRNA^PFC/CaMKIIα^ mice (orange) had lower immobility time as compared with control (grey) in TST (unpaired-T test, *p* = 0.0354, * *p* < 0.05, miRNA group *n* = 12, control group *n* = 13), ns, *p* = 0.100.

To further confirm the anxiolytic effect by miRNA silencing of β2-AR within mPFC CaMKIIα neurons, we used NSFT, a classical test for anxiety assessment. After 24 h fasting, the same group of mice performed the NSFT. We found that the miRNA silencing β2-AR/CaMKIIα mice showed significantly decreased latency to eat compared to the control group (unpair Student T test, Figure 5b, *p* = 0.0049), indicating that the mice with β2-AR knock down in mPFC showed obvious anxiolytic-like behavior. In addition, the total food consumption in homecage after the NSFT showed no significant difference (Figure 5c). TST data showed that miRNA silencing β2-AR/CaMKIIα group demonstrated significantly decreased immobility time compared to control, indicating that miRNA silencing of β2-AR in mPFC induces an antidepression effect (Figure 5d, *p* = 0.0354).

### 2.6. miRNA Silencing of β2-AR within mPFC CaMKIIα Neurons Promotes Social Interaction

**Figure 6:**
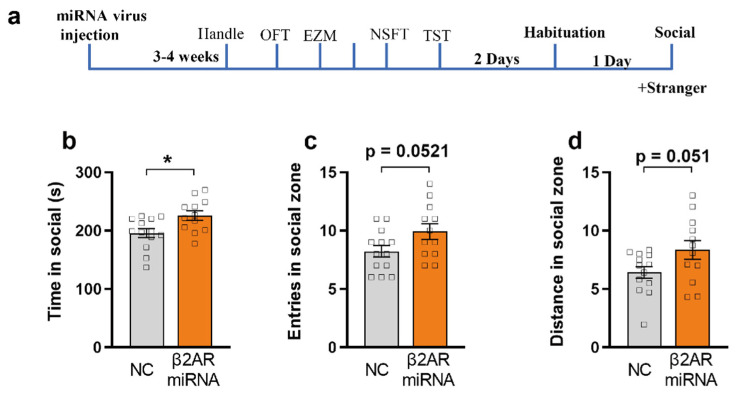
miRNA knock down of β2AR in mPFC CaMKIIα neurons promotes social interaction. (**a**) Behavior timeline of social interaction. (**b**) β2AR-miRNAPFC/CaMKIIα mice (orange) spend more time (*p* = 0.0118) in social as compared with control (grey) (unpaired-T test, * *p* < 0.05). (**c**) β2AR-miRNAPFC/CaMKIIα mice (orange) had no significant difference in entries of social zone as compared with control (grey) (unpaired-T test, *p* = 0.0521). (**d**) β2AR-miRNA^PFC/CaMKIIα^ mice (orange) had no significant difference in travel distance in social as compared with control (grey) (unpaired-T test, *p* = 0.051, miRNA group *n* = 12, control group *n* = 13).

As opto-activation of β2-AR in the mPFC pyramidal neurons induced social-anxiety behavior (Figure 3), we hypothesized that miRNA silencing of β2-AR in mPFC would promote social interaction. Using the social interaction test, we found that both miRNA silencing β2-AR/CaMKIIα and control mice spend similar amounts of time in the social zone in the absence of a novel conspecific (Supplementary Figure S4, unpaired Student’s *t*-test). However, in the presence of a novel mouse, miRNA silencing of β2-AR mice showed a significant increase in the time spent in the social zone compared to the control (Figure 6b, unpaired Student’s *t*-test, *p* = 0.0118). Interestingly, there were no differences in the number of entries and distance in the social zone, suggesting that miRNA silencing β2-AR/CaMKIIα-expressing animals entered the social zone and moved similarly to the control, but remained there for a longer period (Figure 6c,d, *p* = 0.0521 and 0.051).

## 3. Discussion

To investigate how β2-AR in mPFC modulates acute anxiety-like and depression-like behavior, we utilized optogenetics and miRNA silencing to specifically manipulate the β2-AR activity and expression in vivo. In this study, we found that optogenetic activation of β2-AR in mPFC excitatory neurons induced acute anxiety-like behavior in OFT, EZM, and NSFT, and depression-like behavior in TST, and reduced social interaction. Furthermore, miRNA knockdown of β2-AR in mPFC excitatory neurons induced anxiolytic and antidepression effects and promoted social interaction.

Past studies have shown contradictory results regarding the effect of down regulation of β2-AR in anxiety. The Zhu group found that β2-AR knockout mice showed enhanced preference for the closed arm in the elevated plus maze and increased preference in the dark zone of the light dark box test, suggesting that β2-AR knockout increases anxiety level. Furthermore, β2-AR knockout mice exhibited less immobility in TST, indicating that β2-AR knockout induced anxiety- and depression-like behavior [33]. This contradictory result is possible owing to the fact that β2-AR deletion may affect the cardiovascular system, which also correlates with the anxiety level [34,35,36,37]. In the current study, we performed knockdown of β2-AR restricted to mPFC excitatory neurons, resulting in the anxiolytic effect.

The mPFC is one of the key regions in regulating anxiety and mood [19,38]. However, the role of IL and PL in regulating anxiety is still debated. Temporary inactivation of the PL or IL with lidocaine does not affect the anxiety level in OFT [39]. The Suzuki group found that local perfusion of sodium channel activator veratrine in PL instead of IL induces anxiety-like behaviors [40]. The Wang group found that optogenetic inhibition of the PL excitatory neurons initiated anxiety-like behavior, while optogenetic activation of PL excitatory neurons had no effect on anxiety-like behavior [21]. The Adhikari group found that optogenetic inhibition of IL-amydala instead of PL-Amgdala can induce anxiety-like behavior [17]. Activating IL by local perfusion of bicuculline [22] or optogenetic activation of IL excitatory neurons induced anxiety-like behavior in OFT [23]. In our experiment, we optogenetically activated β2-AR in mPFC including PL and IL (Figure 1a,c), resulting in acute anxiety-like behavior (Figure 1 and Figure 2). Also, it is reported that opto-activation of β2-AR instead of channelrhodopsin-2 (ChR2) in dentate gyrus modulates aversive contextual processing, indicating that β2-AR signal activation is different to ChR2 activation [41]. Thus, using β2-AR optogenetics, our study directly supports the notion that β2-AR in mPFC excitatory neurons contribute to acute anxiety-like behavior. Also, our miRNA silencing experiment results (Figure 4, Figure 5 and Figure 6) further support that inhibiting β2-AR in mPFC excitatory neurons induced an anxiolytic effect. It is reported that prefrontal cortical β2-AR activate spike-timing-dependent LTP and enhances fear memory via stimulating postsynaptic cAMP-PKA signaling [24]. Also, selective β2-AR activation can promote adult hippocampal neurogenesis [42], while deletion of β2-AR could enhance place preference for cocaine [33]. Thus, silencing β2-AR in mPFC excitatory neurons may also affect LTP, fear memory, neurogeneisis, and rewarding properties of cocaine.

In addition to excitatory neurons, β2-AR is also expressed in inhibitory neurons, including somatostatin, parvalbumin, calretinin, and calbindin interneurons in the mPFC [43]. The role of the β2-AR in these inhibitory neurons needs to be further studied. The mPFC projects to the BLA, dorsal periaqueductal gray (PAG), nucleus accumbens, and other brain regions linked to mood regulation [38,44]. In vivo inhibition of glutamatergic mPFC projections to dorsal PAG contributes to the behavioral effects of social defeat behavior [44]. Also, mPFC and β-AR play important roles in fear and aversive learning [16,24,45,46]. How the β2-ARs in different cell types of the mPFC are involved in fear and aversive learning remains to be elucidated.

Our data reveals that β2ARs in mPFC regulate anxiety-like behavior, but the cellular and molecular pathway contributing to β2-AR function in anxiety needs to be further investigated. Dong group reported that lactate metabolism regulated by β-arrestin-1 contributes to β2-AR functions in improved memory formation [47]. Also, knock down of astrocytic instead of neuronal β2ARs in the hippocampus impaired formation and consolidation of aversive memory, which can be rescued by L-lactate infusion [48]. Our previous work has shown that L-lactate in anterior cingulate cortex plays important role in rat decision-making ability [49]. Also, arousal-induced cortical lactate release can be reduced by blocking β-AR signaling [50]. In addition, the Hara group found that inhibiting β2AR-β-arrestin-1 signaling pathway could prevents the accumulation of DNA damage in chronic restraint stress in mice, indicating that the β2AR-β-arrestin-1 pathway may contribute to the negative consequences of stress [51]. Thus, further studies are required to elucidate the interaction between β2AR and lactate or other signaling molecules, and how they contribute to anxiety.

Some early literature in humans showed that oral administration of β2-AR antagonists did not appear to be effective in acute anxiety neurosis or chronic anxiety [52,53,54]. This result is possible because that oral administration of β2-AR antagonism may affect the cardiovascular system, which also correlates with anxiety levels [34,35,36,37]. Our study supports that cell-specific silencing β2-AR in mPFC may be a potential therapy for treatment of anxiety-related disorders. Further studies about investigating the anxiolytic effect by using microRNA or AAV therapeutics to inhibit the β2AR system in CNS are needed.

Though we can selectively activate β2-adrenergic signaling in excitatory neurons of the mPFC by using chimeric rhodopsin/β2-AR (Opto-β2-AR) with in vivo optogenetic techniques, this approach comes with some limitations. First, this non physiologic effect via optogenetics could not completely mimic β2-adrenergic signaling. Second, we cannot limit the expression of this recombinant receptor in excitatory neurons that intrinsically express b2-AR.

In summary, using optogenetic β2-AR activation and the miRNA silencing technique, our data support the conclusion that β2-AR in mPFC excitatory neurons play a critical role in acute anxiety-like behavior. However, further studies of these receptors, circuits, and pathways are needed. Our finding provides new insights that β2-AR in the mPFC excitatory neurons regulates anxiety-like behavior and extends our understanding of the development of anxiolytic therapy.

## 4. Materials and Methods

### 4.1. Animal and Ethical Consideration

All the experimental work was carried out on adult male C57BL/6J mice (6–8 weeks old). They were kept in the cages with 24 h access to water and food chow. The animals were maintained in a holding room with a constant room temperature of 25 °C and a 12:12 h light and dark cycle. The experimental trials involving mice surgery were performed in accordance with the guidelines laid down by the Committee on the Use and Care of Animals, City University of Hong Kong and Department of Health, Govt. of Hong Kong SAR, Reference No. (20-17) in DH/HT&A/8/2/5 Pt. 1 and (20–112) in DH/HT&A/8/2/5 Pt. 1.

### 4.2. AAVs Generation

AAV2/9-ef1a-Dio-opto-β2-AR-EYFP (Addgene plasmid No. 20948), AAV2/9-ef1a-Dio-EYFP, AAV2/9-CaMKIIα-cre, were packaged by Taitool Bioscience, Chine. To achieve the knockdown efficiency, the BLOCK-iT Pol II miRNAi expression vector kits were used (Invitrogen, Waltham, MA, USA). Six pre-miRNA sequences for β2AR receptors (β2AR receptors-miRNA) and a negative control sequence (NC-miRNA) were designed using Invitrogen’s RNAi Designer, created, and cloned into a pAAV-CMV-bGI-mCherry-miRNAi vector (Taitool Bioscience, Shanghai, China). The knockdown efficiency was then assessed by co-transfecting EGFP-tagged β2AR receptors with the β2AR receptors miRNA vectors in the human embryonic kidney (HEK293) cell line. The knockdown efficiency was confirmed by a decrease in the fluorescence signal or protein expressed by the EGFP-β2AR receptors vector. The sequence with the highest knockdown efficiency was chosen as follows: β2AR receptors -miRNA, TGCCTTCAATCCTCTTATCTA. The selected oligos were then either cloned into the linearized pAAV-ef1a-Dio-mCherry-miRNAi vector (Taitool Bioscience, China) using T4 DNA ligase. The plasmids were packaged into the AAV2/9 virus by calcium phosphate transfection with capsid and helper vectors on HEK293 cells. The titer of virus was determined by qPCR.

### 4.3. Stereotactic Surgery and Virus Injection

Mice were anaesthetized using ketamine (100.0 mg/kg) and xylazine (8.0 mg/kg). Mice were placed on a stereotactic frame (RWD Instruments). Virus was injected into the mPFC bilaterally (AP, +1.90 mm; ML, ±0.25 mm; DV, −2.8 mm to −2.00 mm from bregma) using a modified microliter syringe (Hamilton) with a 32-gauge needle at a slow rate of 0.1 μL/min. After the injection was completed, the injection needle was left for an additional 9 min before it was slowly withdrawn totally. For the optogenetic experiment, optic fiber (OD 200 nm, inper, China) was placed above mPFC with 10 degrees (AP, +1.90 mm; ML, ±1.00 mm; DV, −2.10 mm from bregma), and secured to the skull with dental cement (C&B Metabond, Parkell, Edgwood, NY, USA). Then, mice were put to a heat pad to recover from anesthesia.

The following viral vectors were used: AAV2/9-ef1a-Dio-opto-β2-AR-EYFP (Addgene plasmid No. 20948; packaged by Taitool Bioscience, China), AAV2/9-ef1a-Dio-EYFP (diluted titer: 5.00 × 10^12^ v.g./mL, 0.10 μL, bilateral into mPFC, taitool Bioscience), AAV2/9-CaMKIIα-cre (diluted titer: 6.00 × 10^12^ v.g./mL, 0.10 μL, bilateral into mPFC, taitool Bioscience), AAV2/9-ef1a-Dio-miRNA-β2-AR-mcherry, AAV2/9-ef1a-Dio-NC-mcherry (titer: 5.00 × 10^12^ v.g./mL, 0.10 μL, bilateral into mPFC, taitool Bioscience). All viral vectors were aliquoted and stored at −80 °C until use.

### 4.4. Immunohistochemistry

After all the experiments, mice were perfused with PBS followed by 4% paraformaldehyde (PFA) in PBS. The brains were extracted, postfixed overnight in 4% PFA at 4 °C, and cryoprotected in 30% sucrose. Brains were sectioned to a thickness of 30 mm using a sliding freezing microtome (Leica SM2010R) and preserved in a cryoprotectant 30% ethylene glycol, in PBS). Free-floating sections were washed in PBS, incubated for 1 hr in blocking solution (10% normal goat serum (NGS) and 0.3% Triton X-100 in PBS), and incubated overnight at 4 °C with primary antibodies (rabbit anti-β2-AR, Thermo Scientific, Alomone Labs, 1:500; Mouse anti-CaMKIIα, 1:500; rabbit anti c-fos, Synaptic Systems, 1:500) in 0.1% Triton and 3% NGS in PBS. Sections were then washed with PBS 4 times and incubated for 2.5 h at room temperature with secondary antibodies (goal anti-rabbit, Alexa Fluor 594, 1:300; goal anti-mouse, Alexa Fluor 488, 1:300; goal anti-mouse, Alexa Fluor 594, 1:500; goal anti-rabbit, Alexa Fluor 647, 1:300; goal anti-rabbit, Alexa Fluor 405, 1:300) in PBS. Finally, sections were washed in PBS 4 times, mounted on slides and sealed with mounting medium (Fluoromount-G, eBioscience, San-Diego, CA, USA). Mounted slides were imaged using an inverted laser scanning confocal microscope (LSM 880; Carl Zeiss, Oberkochen, Germany).

### 4.5. Behavior

For animal behavior study, all behavioral tests were performed in mice 9–12-week-old. We have 19 mice for the optogenetic experiment (10 for control group, 9 for opto-β2AR group), and another 25 mice for microRNA silencing experiment (13 for control, 12 for miRNA group). During optogenetic experiment, the optic fiber was connected to a laser source using an optic fiber sleeve. Opto-β2AR mice and control mice underwent the same procedure and received the same intensity of laser stimulation (473 nm, 10 Hz, 10 ms width (10% duty), 8–10 mW power; no seizure-like behavior observed for all the stimulation) continuously via optic fiber [55], during the whole process of each behavior test. The overall duration of the opotogenetic stimulation in OFT, EZM, and social interaction test is 6 min, while duration of opto-activation in the NSFT and TST is 5 min., during the whole process of each behavior test.

#### 4.5.1. Open-Field Test (OFT)

The test was conducted in a square open arena (500 × 500 × 500 mm) to quantify animals’ locomotor activity and anxiety-like behavior. The mice were positioned in the arena for 6 min under natural light condition (160 lux). For the optogenetic experiment, mice were kept in the OFT for extra 6 mice after optogenetic activation. The arena was cleaned with 30% ethanol between each test. The center area of OFT was defined as a square which occupied 50% the total area of the arena. The exploration distance, time in center area, and entries of the center zone were measured using Anymaze (Stoelting Co., Wood Dale, IL, USA) tracking software.

#### 4.5.2. Elevated Zero Maze (EZM)

The EZM apparatus was made of grey plastic, 2000 mm in circumference, comprised of four 500 mm sections (two closed and two opened) to test anxiety-like behavior in mice. The apparatus was elevated 520 mm above the floor, and it had a path width of 45 mm with a 4 mm lip on the open section. The test was performed for 6 min under a condition of natural light (160 lux). For optogenetic experiment, mice will be kept in the EZM for an extra 6 mice after optogenetic activation. The exploration distance, time in the open zone, and entries to the open zone were measured using Anymaze (Stoelting Co., USA) tracking software.

#### 4.5.3. Novelty-Suppressed Feeding Test (NSFT)

The test was performed in a round open field arena (diameter 400 mm, height 400 mm). Food pellets were positioned in the center of the arena. The amount of food was about 5 g. For this experiment, mice were food restricted for a period of 24 h. After food restriction, the experiment was carried on by placing the animals in the corner of arena and measuring the time to approach and eat a pellet of food. The test duration was 6 min. Latency to approach and eat the food pellet were used to measure anxiety-like behavior using Anymaze (Stoelting Co., USA) tracking software. Subsequently, animals were allowed to eat the food pellet for additional 5 min in their home cage. The total food pellet was weighed to calculate the amount of food intake.

#### 4.5.4. Tail-Suspension Test (TST)

All mice were subjected to the tail-suspension test for a duration of 5 min. The mice were suspended by hanging their tails with adhesive plaster in an individual compartment of 400 mm height away from the floor. The immobility time were measured when mice were entirely motionless to test behavioral despair, which is typical anxiety-like behavior using Anymaze (Stoelting Co., USA) tracking software.

#### 4.5.5. Social Interaction Behaviors

The social interaction behavior was performed in a rectangular arena (length 600 mm, width 200 mm, height 240 mm) containing three chambers. Each chamber was 200 × 200 mm, divided by plastic walls with an open middle section to allow mice to freely move from between chambers. Two identical cage-like cylindrical containers were positioned in the middle of right and left chamber, one for each side. On the first day of this test, testing mice were put into the center of the arena for a 6 min duration of habituation, allowing them to freely move from each chamber; 24 h later, on the second testing day, a wildtype c57 mouse that had never approached the testing mice were put inside one of the containers (social zone) as a stranger. Testing mice were placed in the center of the middle chamber. Stranger mice were changed between each trial and the arena were cleaned by 30% ethanol. Each test duration was 6 min and the exploration distance, time in the social zone, and entries of social zone were measured using Anymaze (Stoelting Co., USA) tracking software.

### 4.6. Quantification and Statistical Analysis

All data are expressed as mean ± SEM. Statistical significance was taken as * *p* < 0.05, ** *p* < 0.01, *** *p* < 0.001, as determined by Student’s *t*-test (unpaired). Statistical analyses were performed in GraphPad Prism 9 (Graph Pad, San Diego, CA, USA).

## Data Availability

The data supporting the current study have not been deposited to any public repository but are available from the corresponding author on request. This study did not generate code.

## Supplementary Materials

The following supporting information can be downloaded at: https://www.mdpi.com/article/10.3390/ijms23105578/s1.
