# Supplementary material for: β2-Adrenoceptors in the Medial Prefrontal Cortex Excitatory Neurons Regulate Anxiety-like Behavior in Mice
Source: Int J Mol Sci. 2022 May 17;23(10):5578. doi: 10.3390/ijms23105578 (PMC9145949; doi:10.3390/ijms23105578)
Supplement: Supplementary file 1 [file ijms-23-05578-s001.zip › ijms-1664912-supplementary.pdf]

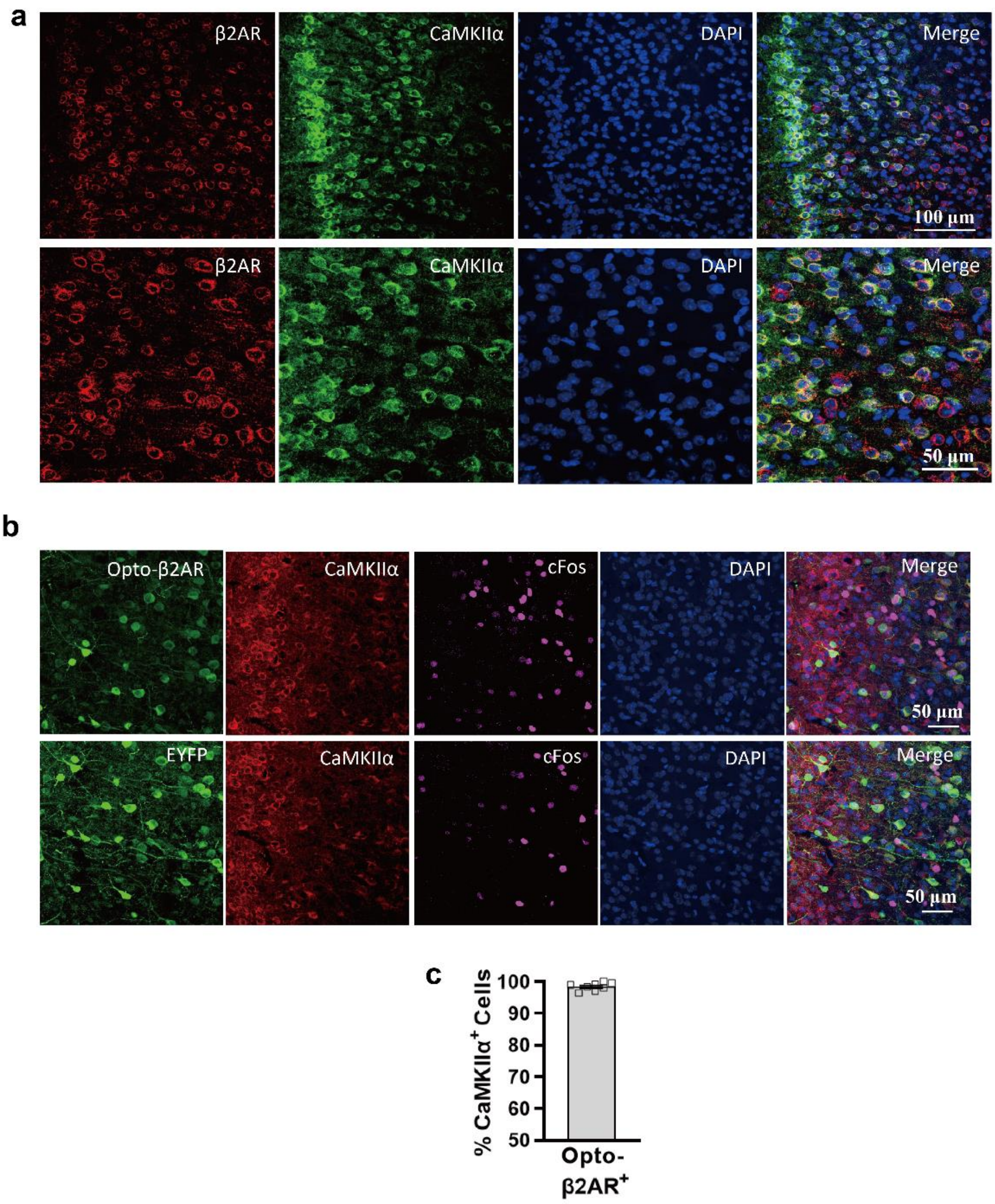

Supplemental Figure S1. β2-AR is abundantly present in CaMKIIα positive neurons in the mPFC, and opto-β2-AR are highly co-expressed with CaMKIIα and cFos.

(a) Immunofluorescence images showed that β2-AR (red, Alomone Labs, #AAR-016) is abundantly present in CaMKIIα positive neurons (green) in the mPFC. Up panel is low magnification images, down panel is high magnification images.

(b) Immunofluorescence images showed that opto-β2-AR (green) or EYFP (green) and CaMKIIα (red) are highly co-expressed in the mPFC. Up panel is opto-β2AR group, down panel is EYFP control group.

(c) Quantification of percentage of total opto-β2AR positive cells co-expressed with CaMKIIα (n = 4, 2 slices for each mouse).

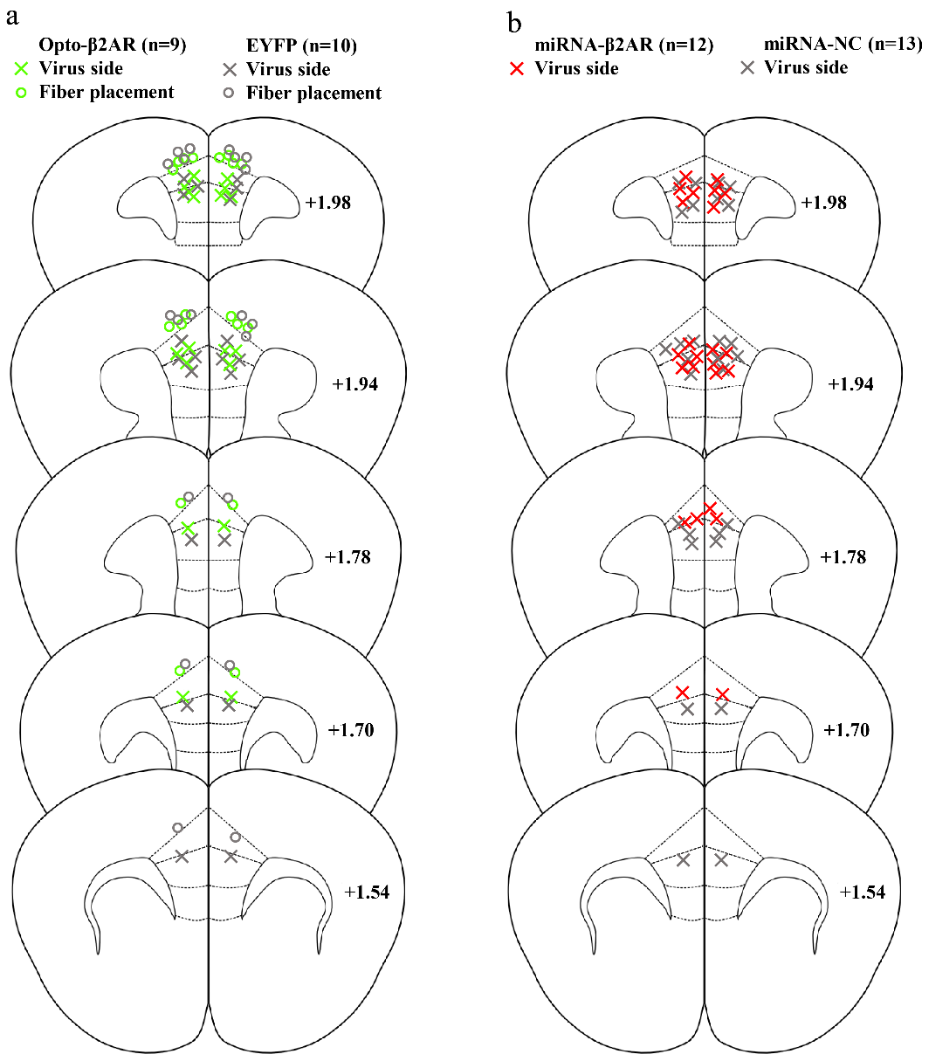

Supplemental Figure S2. Graphical summary of the site of injection of AAVs for the individual animals for each experiment.

(a) Virus injection side (crosses) and optic fiber placement (circles) of opto-β2AR (green, n = 9) and EYFP (gray, n = 10) in mPFC (the graphical figure is adapted to (Felix-Ortiz et al., 2016)).

(b) Virus injection side (crosses) miRNA-β2AR (red, n = 12) and miRNA-NC (gray, n = 13).

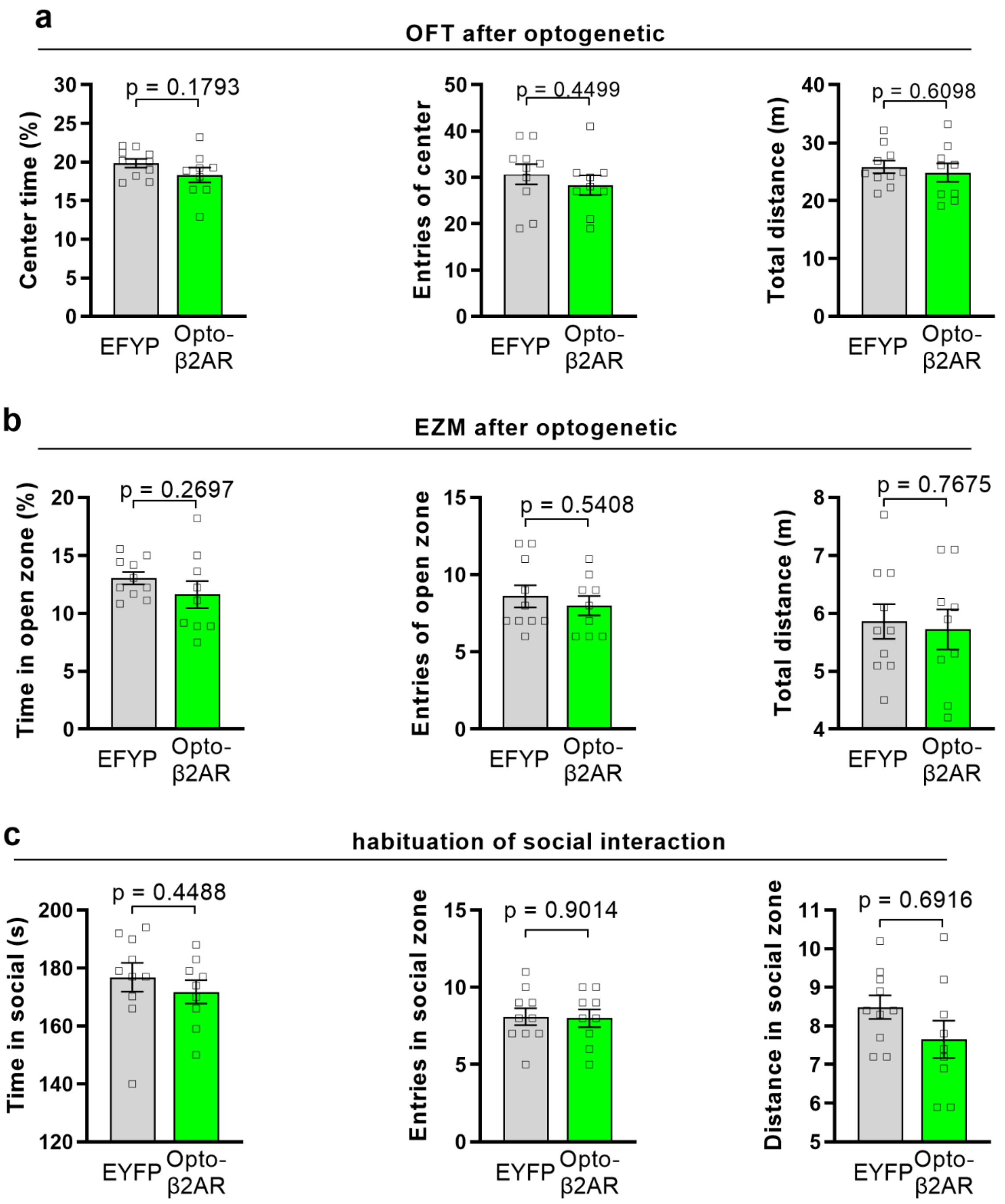

Supplemental Figure S3. Opto-β2AR mice and control mice have no significant difference in anxiety level after optogenetic and place preference during habituation of social interaction.

(a) Opto-β2AR$^{PFC/CaMKii}$ mice (green) showed no significant difference in time in center, entries in center, and total activity after optogenetic in OFT, compared with control (grey) (unpaired-T test).

(b) Opto-β2AR$^{PFC/CaMKii}$ mice (green) showed no significant difference in time, entries in open zone, and total activity after optogenetic in EZM, compared with control (grey) (unpaired-T test).

(c) Opto-β2AR$^{PFC/CaMKii}$ mice (green) showed no significant difference in time in social zone, entries in social zone, and distance in social zone during habituation of social interaction test, compared with control (grey) (unpaired-T test) (Opto-β2AR group, n = 9; control group, n =10).

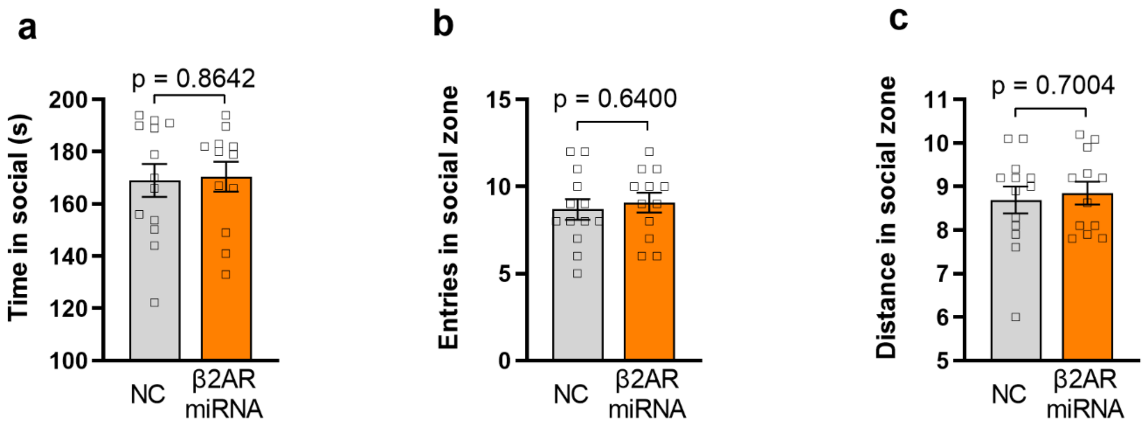

Supplemental Figure S4. miRNA-β2AR mice and control mice have no significant difference in the place preference during habituation of social interaction.

(a) β2AR-miRNA$^{PFC/CaMKii}$ mice (orange) spent no significant different time in habituation social (no mouse) zone as compared with control (grey) (unpaired-T test, p = 0.8642).

(b) β2AR-miRNA$^{PFC/CaMKii}$ mice (orange) had no significant different entries in habituation social zone as compared with control (grey) (unpaired-T test, p = 0.6400).

(c) β2AR-miRNA$^{PFC/CaMKii}$ mice (orange) had no significant different travel distance in habituation social zone as compared with control (grey) (unpaired-T test, P = 0.7004).

(miRNA-β2AR group, n = 12; control group, n =13).
